# Blood as Food

**Published:** 1860-04

**Authors:** 


*Employment of Blood as Food.*—We certainly live in an age of projects and suggestions, and we must be very blind to what is going on in transatlantic countries, if we imagine that cisatlantic ingenuity is only employed in producing innovations upon the customs and habits of the world. In all honesty, this *is* a fast age. Everything is made *to pay*, in some way or other. Inaction is considered the great crime, and less than six per cent. a poor return for any venture. Hear the French journalist, Guérolt, in the *Opinion Nationale*, as he talks of the new pamphlet of Dr. Steinroth—from the sober land of Germany—entitled *De la chair coulante et de son exploitation rationnelle.*

"The *chair coulante* (liquid flesh) is the blood. The German economist pretends that, despite our advanced civilization, the agricultural arts are yet in their infancy, and that real economical zootechny is yet to be created. We have dairies which abundantly furnish all European countries with milk; but meat is deficient, and its price becomes higher and higher every day. There are, moreover, in Europe three or four times as many beeves as cows. Let us, then, establish *laiteries de chair*, (dairies of blood;) let us obtain from animals their blood as we do their milk. Blood contains no gelatin, and but little fatty matter—still it is composed of all the other substances which make flesh *par excellence* the nutrient food. It is true, one cannot bleed beeves as often as we can milk cows, yet bleeding can be repeated weekly, and for several years, on the same animal without injuring its health, and without removing the flesh, which we shall get afterwards. Thus we may obtain from a beef, a sheep, or a hog, three or four times as much alimentary material as we obtain now by slaughtering them. The blood could be eaten crude or cooked, pure or mixed with milk, or introduced into vegetable food, especially bread and pastries."

"The proceeding is not new. In Sweden, blood is employed in the preparation of a species of biscuit; in Ireland, (*sic!*) the poor frequently bleed beeves and cows to procure that substantial food which is otherwise denied them. In Africa the custom of using blood is quite extensive, and blood is the principal nourishment of a large number of people; like the Adjeba of the basin of Sobat, who only rear large herds for the purpose of subjecting them to repeated bleedings."

Guérolt adds: "We doubt, notwithstanding the arguments employed, whether this system of economical zoology will ever be adopted in France. The dairies of *chair coulantes* may be very highly appreciated among the negroes of Soudan, but in Europe there is an instinctive repugnance to the use of blood."

The editor of *L'Union Médicale* says this repugnance is not so great as might be supposed. *Boudin* (blood-pudding)—a very popular food —is almost exclusively made of hog's blood. In all the central provinces of France, the blood of almost all the fowls killed is carefully preserved, and, by means of a very simple mode of preparation, is fitted for the table. We understand how one could advantageously introduce, as food, blood of animals slaughtered by the butcher, which is now employed for other purposes, but we object to the proposition of Steinroth; it is a barbarous and cruel practice.

Who will make the effort to introduce blood on this side of the great ocean; who will establish a *blood dairy*? Shall the Rhine take the lead in this matter? Oh that Soyer were now alive! How many dainty dishes would he *compose*, the central and attractive feature in each of which should be *chair coulant!* We shall keep our eyes open to report the progress of the blood movement, although we fear some friends of the animal kingdom may strongly oppose the idea of keeping herds of beeves simply for the purpose of performing weekly venesection upon them. L. H. S.

